# Circadian Genes Expression Patterns in Disorders Due to Enzyme Deficiencies in the Heme Biosynthetic Pathway

**DOI:** 10.3390/biomedicines10123198

**Published:** 2022-12-09

**Authors:** Maria Savino, Claudio Carmine Guida, Maria Nardella, Emanuele Murgo, Bartolomeo Augello, Giuseppe Merla, Salvatore De Cosmo, Antonio Fernando Savino, Roberto Tarquini, Francesco Cei, Filippo Aucella, Gianluigi Mazzoccoli

**Affiliations:** 1 Interregional Reference Center for Porphyria, Fondazione IRCCS “Casa Sollievo della Sofferenza”, 71013 San Giovanni Rotondo, Italy; 2 Laboratory of Clinical Chemistry, Fondazione IRCCS “Casa Sollievo della Sofferenza”, 71013 San Giovanni Rotondo, Italy; 3 Department of Medical Sciences, Division of Nephrology, Fondazione IRCCS “Casa Sollievo della Sofferenza”, 71013 San Giovanni Rotondo, Italy; 4 Department of Medical Sciences, Division of Internal Medicine and Chronobiology Laboratory, Fondazione IRCCS “Casa Sollievo della Sofferenza”, 71013 San Giovanni Rotondo, Italy; 5 Division of Medical Genetics, Fondazione IRCCS “Casa Sollievo della Sofferenza”, 71013 San Giovanni Rotondo, Italy; 6 Department of Molecular Medicine and Medical Biotechnology, Federico II University, 80121 Naples, Italy; 7 Laboratory of Regulatory and Functional Genomics, Fondazione IRCCS “Casa Sollievo della Sofferenza”, 71013 San Giovanni Rotondo, Italy; 8 Division of Internal Medicine I, Regional Reference Center for Porphyria, San Giuseppe Hospital, 50053 Empoli, Italy

**Keywords:** heme, porphyria, circadian, rhythm, biological clock

## Abstract

Heme is a member of the porphyrins family of cyclic tetrapyrroles and influences various cell processes and signalling pathways. Enzyme deficiencies in the heme biosynthetic pathway provoke rare human inherited metabolic diseases called porphyrias. Protein levels and activity of enzymes involved in the heme biosynthetic pathway and especially 5′-Aminolevulinate Synthase 1 are featured by 24-h rhythmic oscillations driven by the biological clock. Heme biosynthesis and circadian pathways intermingle with mutual modulatory roles. Notably, heme is a ligand of important cogs of the molecular clockwork, which upon heme binding recruit co-repressors and inhibit the transcription of numerous genes enriching metabolic pathways and encoding functional proteins bringing on crucial cell processes. Herein, we assessed mRNA levels of circadian genes in patients suffering from porphyrias and found several modifications of core clock genes and clock-controlled genes expression, associated with metabolic and electrolytic changes. Overall, our results show an altered expression of circadian genes accompanying heme biosynthesis disorders and confirm the need to deepen the knowledge of the mechanisms through which the alteration of the circadian clock circuitry could take part in determining signs and symptoms of porphyria patients and then again could represent a target for innovative therapeutic strategies.

## 1. Introduction

Deficiency of enzymes of the heme biosynthetic pathway causes a category of human metabolic diseases called porphyrias, characterized by different modes of inheritance and clinical manifestations, with signs and symptoms manifesting in different organ systems [1,2,3]. Porphyrias are associated with build-up and undue excretion of heme pathway intermediates, such as the heme precursors 5-aminolevulinic acid (ALA) and porphobilinogen (PBG) and their oxidized products, such as porphirins [4,5]. They are classified in acute porphyrias (affecting nervous system and skin) and cutaneous porphyrias (affecting mainly the skin) [6]. Acute porphyrias comprise acute intermittent porphyria, variegate porphyria, hereditary coproporphyria, and delta-aminolevulinic acid dehydratase deficiency porphyria [6]. Cutaneous porphyrias include porphyria cutanea tarda, erythropoietic protoporphyria, X-linked protoporphyria, congenital erythropoietic porphyria, and hepatoerythropoietic porphyria [6]. Acute intermittent porphyria (AIP) is inherited in an autosomal dominant manner and mutations cause partial deficiency of PBG deaminase, the third enzyme of the heme biosynthetic pathway performing the polymerization of the monopyrrole PBG into hydroxymethylbilane. ALA and PBG accumulate during overt crises, which manifests with neuro-visceral symptoms intensified by some drugs, hormones, and nutritional changes. AIP treatment relies on intravenous injection of ferric chloride salt of heme (hemin) or carbohydrate loading for mild attacks or when hemin is unobtainable [7,8]. In general, porphyrias are inherited diseases, while porphyria cutanea is caused by an acquired enzymatic deficiency in liver, although an inherited deficiency is a predisposing factor in some cases (Yasuda 2018) [9]. ALAS1 (5′-Aminolevulinate Synthase 1), the rate limiting enzyme in heme biosynthesis, oscillates with circadian rhythmicity driven by the biological clock [10,11,12,13]. Biological processes show variations in the temporal dimension that may be sporadic or recurrent and, in this case, may be rhythmic or arrhythmic. Periodic variations recurring with a frequency of one cycle in approximately 24 h are defined as circadian. Circadian rhythmicity characterizes the function of body systems that allow homeostasis maintenance, and among these, the metabolic pathways are preeminent. Derangement of customary circadian rhythmicity underlies pathophysiological mechanisms in several metabolic [14,15,16,17,18,19,20,21,22]. Circadian rhythms are endogenous cycles driven by molecular clockworks ticking in mammals through transcriptional/translational feedback loops operated by a positive limb, working through the transcriptional activators ARNTL/ARTL2 and CLOCK (or NPAS2) which are complex and activate a negative limb, with Period (PER1, PER2, PER3) and Cryptochrome (CRY1, CRY2) proteins that in turn inhibit the transactivation complex and close the loop [23,24]. The circadian proteins must be post-translationally modified, mainly phosphorylated by caseinkinases, and tagged for proteasomal degradation or acetylated/deacetylated by sirtuins to allow unrelenting and properly self-sustained clock functioning [25,26]. The ARNTL/ARNTL2-CLOCK complex activates also the expression of the nuclear receptors RORs (α,β,γ) and REV-ERBs (α,β), which drive the expression of ARNTL. Besides, the ARNTL/ARTL2-CLOCK (or NPAS2) complex also activates first order clock-controlled genes (DBP, HLF, TEF, NFIL3), driving the expression of thousands of genes involved in metabolic pathways and crucial cell processes [27]. 

Heme modulates numerous cell processes related to oxygen sensing/transport, electron transfer-mediated biochemical reactions, and signalling pathways [28]. Remarkably, heme is a ligand of REV-ERBs, which upon heme binding recruit the co-repressor NCoR with REV-ERB-NCoR complex stabilization, inhibit the transcription of target genes involved in glucose and lipid metabolism, and modulate ARNTL expression as well as the expression of ARNTL-driven circadian genes and their encoded proteins [29]. Besides, heme works as a regulatory ligand in humans through a heme-regulatory SC(841)PA motif within the C terminus of Per2, and, interacting with its stabilizing counterpart cryptochrome, reduces Per2 stability binding solely to ferric heme and consequently performing as a redox sensor capable to modulate circadian gene expression and period length [30]. 

On these premises, we aimed to evaluate the expression of core clock genes and clock-controlled genes in humans affected by disorders due to enzymatic deficiency/dysfunction in the pathway of heme biosynthesis. 

## 2. Materials and Methods

### 2.1. Patients

In the present study, thirty-four patients with different forms of porphyria were enrolled: twenty-one patients affected by acute intermittent porphyria (AIP), seven patients affected by hereditary coproporphyria (HCP), four patients affected by porphyria cutanea tarda (PCT), and two patients affected by congenital erythropoietic porphyria (CEP). Some of them (seventeen) presented clinical symptoms and alterations of biochemical analysis, and for some of them, diagnosis was confirmed by genetic analysis, while seventeen asymptomatic family members were enrolled for genetic testing. In addition, fifteen healthy blood donors were enrolled as controls, as they are considered appropriate candidates for studies of genetic respect to a random sample of the population [31].

Several laboratory tests are available for diagnosis of porphyria [32]. In the present study, the laboratory diagnostic procedures were performed using two important sequential steps: biochemical analysis and molecular analysis.

### 2.2. Biochemical Analysis

The first step for establishing the diagnosis of porphyria comprises the following biochemical analyses: plasma porphyrin scan, ALA and PBG determination, measurement of urine porphyrins, and porphobilinogen deaminase (PBGD) enzyme activity measurement. The fluorometric emission scanning (using excitation at 405 nm) of plasma samples, simply diluted five-fold in phosphate-buffered saline, allows the differentiation of three conditions according to their porphyrin content. The emission maximum at 626–628 nm is a specific finding in variegate porphyria, while in erythropoietic protoporphyria a characteristic peak is found at 636 nm. A fluorescence emission maximum at 618–622 nm corresponds to a third group that includes normal subjects, non-porphyria patients and patients suffering from acute intermittent porphyria, hereditary coproporphyria, congenital erythropoietic porphyria, and porphyria cutanea tarda [33].

ALA and PBG are commonly quantified after purification, from a spot urine sample, using the commercially available anion-exchange and cation-exchange columns for photometric determination of urinary 5-ALA and PBG (ClinEasy^®^ Complete Kit for ALA/PBG in Urine, Recipe GmbH, Munich, Germany). This test is revealing for AIP diagnosis. The measurement of urine porphyrins was performed using commercially available anion-exchange and cation-exchange columns for photometric determination of urinary porphyrins (ClinEasy^®^ Complete Kit for Total Porphyrins in Urine, Recipe GmbH, Munich, Germany). Although various methods have been developed for the analysis of porphyrins, reverse-phase high-pressure liquid chromatography (HPLC) coupled with fluorescence detection has become the gold standard method in this regard to determine URO, HEPTA, HEXA, PENTA, and COPRO I and III concentrations in urine. The complete separation of these heme synthesis intermediates is important for diagnosing certain types of porphyrias. The PBGD enzyme activity measurement is an enzymatic assay used for suspected diagnosis AIP and in our laboratory is performed for the biochemical confirmation of this form of porphyria. The measurement of PBGD activity is based on the measurement of the rate of synthesis of uroporphyrin from PBG in incubated, lysed erythrocytes (PBGDW Porphobilinogen Deaminase, Washed Erythrocytes, Mayo Clinic Laboratories, Rochester, MN, USA).

### 2.3. Molecular Analyses

According to clinical symptoms and the routine biochemical procedure, the diagnosis of one of the different forms of porphyria is subsequently confirmed by the sequence analysis of one of the responsible genes for each type. On the basis of diagnostic suspicion, the propositus and successively the first-degree relatives were analyzed. In particular, the *HMBS* gene was analyzed in twenty-one patients suspected for AIP, seven patients with CPO were analyzed for *CPOX* gene mutations, four CTP patients were screened for *UROD* mutations, and the gene *UROS* was sequenced in two propositus with both clinical and biochemical evidence of PEC. For each gene, all the coding regions and exon/intron boundaries were analyzed using direct sequencing by Sanger method. A sequence analysis of *HMBS* (NM_000190.3), *CPOX* (NM_000097.5), *UROD* (NM_000374.4) and *UROS* (NM_000375.2) genes allowed to determinate three previously described variant and eight novel variations.

### 2.4. HMBS Variants

In twenty-one AIP patients enrolled in this study, a five-point mutation in *HMBS* gene was identified in twelve propositus and family members. No variation was found in the remaining nine propositus.

A well-represented guanine deletion at position 181 in exon 5 of *HMBS* gene, causative of premature truncation of the protein (96 aminoacids instead of 361 aminoacids) was identified in a symptomatic woman. Two splicing variations in *HMBS* gene were identified: c.652-2delA (IVS12-2delA) and c.772-3C > G (IVS 13-3C > G). A previously described deletion of an adenine in position -2 of acceptor site of exon 12 was detected in a propositus and in his two siblings and in another unrelated proband. In the acceptor site of exon 13 a cytosine to guanine substitution c.772-3C > G was detected in a woman with severe symptoms of porphyria. 

In the family of AIP 09-018 a previously described missense variation (c.580C > T) was detected in the propositus and in three asymptomatic family members.

The two brothers enrolled in this study, with only one of them symptomatic, presented a novel six-nucleotide TACCCG deletion in exon 3 of *HMBS* gene at positions 72 and 77 of the cDNA (numbered from the translation initiation codon ATG), that results in a deletion of two amino acids in the protein which causes the p.Thr25_Arg26del, inherited from the father (not enrolled in this study). The same mutation was not found in healthy unrelated individuals. Threonine-Arginine at position 25–26 is highly conserved among species, and the p. Thr25_Arg26del was predictive of a significant conformational change.

### 2.5. CPOX Variants

Three missense substitutions in *CPOX* gene were identified in three hereditary porphyrias propositus. A guanine to timine substitution was detected at position 613 in exon 2 leading to the amino acid substitution p.205V > L in two symptomatic brothers and in their asymptomatic sister and mother. The c.395C > T transition in exon 1 of *CPOX* gene was responsible for the p.132A > V substitution in a twenty-year-old proband. This variation was maternally inherited from the maternal grandmother. A c.148C > T transition causative for p.50P > S was detected in only symptomatic women. It is difficult to determine, in the absence of functional data, whether p.205V > L, p.132A > V and p.50P > S represent pathogenic mutations. However, the three missense mutations affect residues that are highly conserved across species and were absent in 100 control chromosomes, therefore these substitutions likely determine a conformational change affecting the signaling pathway.

### 2.6. UROD Variant

In *UROD* gene, two novel variants are described: an adenine to guanine substitution at position 1000 and a deletion of three nucleotide in exon 4. In exon 10 of *UROD* gene, a missense variation causative of p.I334V was found in three asymptomatic siblings of a patient. Three nucleotide deletions found in exon 4 of *UROD* gene, c. 246-248delCAT, is causative of deletion of isoleucine at position 82 of the protein.

Finally, a diagnosis of PEC, a rare and severe recessively transmitted porphyria, was confirmed in two Pakistani brothers by identification of a splicing site variation c.660+4delA in donor splice site of exon 9. The deletion at homozygous status was inherited from consanguineous parents. The characterization of splicing by reverse transcription and sequencing allowed to demonstrate the deletion of 99 nucleotides corresponding to *UROS* gene exon 9 removing during mRNA transcription.

### 2.7. Real-Time Quantitative Reverse Transcription Polymerase Chain Reaction

We used snapshot mRNA extracted from peripheral blood mononuclear cells (PBMC) sampled at the same time-of-day (09:00 a.m.). Total RNA from patients and controls was extracted using the RNeasy^®^ Mini Kit (QIAGEN) and subsequently digested by DNase I. cDNA was synthesized from 100 ng total RNA with Quantifast RT-PCR kit (QIAGEN). For real-time PCR, we used the Human QuantiTec Primers Assay (SYBR Green QuantiTect Primers Assay; QIAGEN) (Supplementary Table S1). All qPCRs were performed in a 10 μL final volume, with three replicates per sample. Reactions were set up in 96-well plates using a 7900HT Real-Time PCR System (Applied Biosystems, Foster City, CA, USA). Expression levels of the target genes were normalized using the housekeeping control gene GAPDH. The mRNA amount of each target gene relative to control gene was calculated through the comparative Ct method (i.e., the 2(−ΔΔCt) method).

### 2.8. Statistical Analysis

Porphyrias are rare diseases (defined as a condition that affects less than 1 in 2000 people in the European Union or less than 200.000 people in the USA) [34], hence, we planned to enroll and test a small sample size [35]. Sample size estimates for hypothesis testing based on achieving statistical power = 0.8 with effect size = 0.8, β = 0.2 and α = 0.05 projected as a minimum 15 subjects per group [36]. To upkeep statistical power and numerosity, we unified patients with CEP and PCT in one group to confront AIP patients, HCP patients, and control subjects. We report categorical variables as frequencies and percentages, and continuous variables as median and interquartile ranges. Circadian gene expression levels were calculated using the 2-ΔΔCt formula and reported as median, 25th percentile (Q1) and 75th percentile (Q3). For patients with multiple evaluations of clinical/biochemical data at different acquisition timings, we considered arithmetic means of the reported values for statistical evaluation. For our analyses, we used nonparametric statistics. Differences between groups for categorical variables were tested with the Fisher exact test and Cochran’s Q test. Hypotheses regarding differences among two groups for continuous variables were tested by means of the Mann–Whitney rank sum test; we used the Kruskal–Wallis one-way analysis of variance to test differences among multiple groups for continuous variables. For the evaluation of the relationships between circadian gene expression levels and clinical/biochemical variables of patients affected by porphyrias, we calculated Spearman’s rank correlation coefficients (r) considering the gene expression level IQRs of controls as reference values, and we tested differences between patients affected by porphyrias with gene expression levels equal to or higher than the 75th percentile (over-expression) and patients affected by porphyrias with gene expression levels equal to or lower than the 25th percentile (down-regulation). Correlations among circadian gene expression levels were evaluated calculating Spearman’s rank correlation coefficients (r). 

We performed a retrospective study and some clinical and laboratory data could be lost. We reported data and a univariate analysis for all the variables included in the study. List-wise deletion of missing values was performed. We did not test differences between variables that had the number of tested cases inferior to 60% of the total cases. For all the analyses, a *p*-value below 0.05 was considered statistically significant. All the statistical analyses were performed using MedCalc statistical software (MedCalc Software, Acacialaan 22, Ostend, 8400, Belgium).

## 3. Results

Of the 34 patients affected by porphyrias and consecutively enrolled in our study, 14 (41%) were males, and the median age was 46 (IQR 29–54) years. Table 1 shows characteristics and clinical/biochemical features and Table 2 shows gene and protein mutations found in the patients affected by porphyrias enrolled in our study. Most patients had values in the normal range for the tested variables, with the predictable exception of blood, urinary, and fecal porphyrins and metabolites, which resulted as elevated in almost all the tested patients. Only one patient had a positive urine culture for *Escherichia coli*; another one was positive for S antigen of Hepatitis B and one for Hepatitis C antibodies. One patient was obese and three were overweight, and five had hypercholesterolemia. One patient showed subclinical Hashimoto thyroiditis. Three patients showed elevated transaminases values. Three patients were anemic and five had low ferritin levels; two had a low platelets count. Four patients showed a mild degree of hyperparathyroidism.

**Figure 1:**
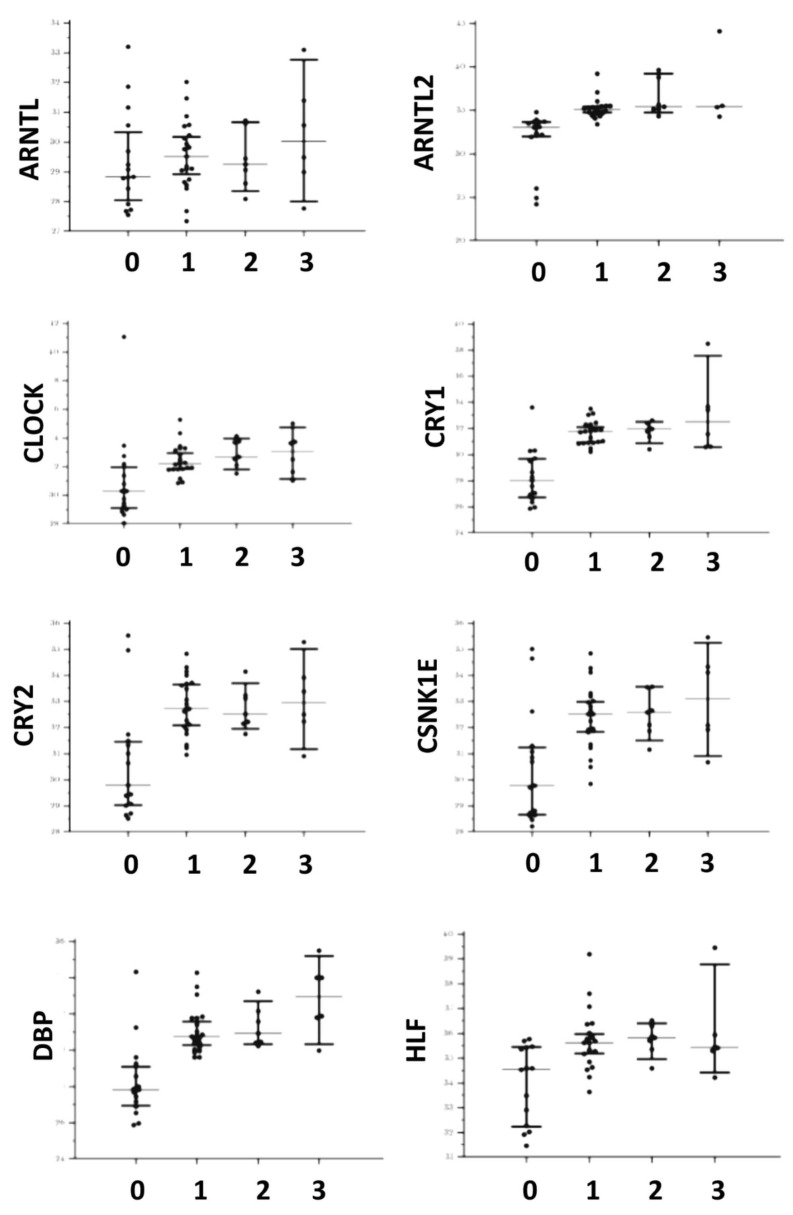
Distributions of gene expression in both controls and affected patients for ARNTL, ARNTL2, CLOCK, CRY1, CRY2, CSNK1E, DBP, and HLF. The central line corresponds to the median, upper, and lower bars to the IQR. For the X axis label, 0 corresponds to controls, 1 to AIP, 2 to HCP, and 3 to CEP and PCT.

**Figure 2:**
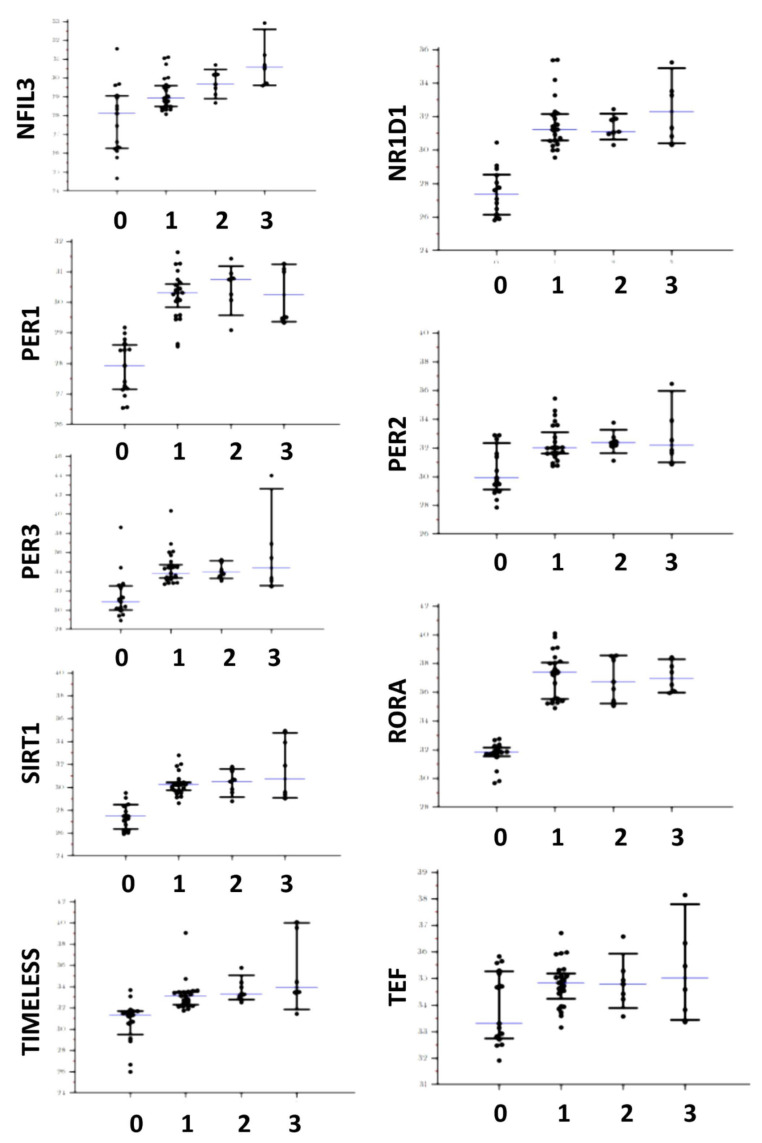
Distributions of gene expression in both controls and affected patients for NFIL3, NR1D1, PER1, PER2, PER3, RORA, SIRT1, TEF, and TIMELESS. The central line corresponds to the median, upper, and lower bars to the IQR. For the X axis label, 0 corresponds to controls, 1 to AIP, 2 to HCP, and 3 to CEP and PCT.

Differences of circadian genes expression tested with the Kruskal–Wallis one-way analysis of variance were detailed in Table 3 and distributions are shown in Figure 1 and Figure 2. With the exception of ARNTL and TEF, all genes appeared significantly over-expressed in patients affected by all the tested types of porphyrias (both acute and cutaneous forms) with respect to controls. In addition, DBP and NFIL3 showed a significantly higher expression in patients affected by CEP and PCT with respect to AIP. No gene appeared down-regulated with respect to the controls. As shown in Table 4, ARNTL, CLOCK, CRY1, CRY2, CSNK1E, DBP NFIL3, NR1D1, PER2, and SIRT1 showed significantly lower expression in symptomatic patients. No significant difference was found for ARNTL2, HLF, PER1, PER3, RORA, TEF, and TIMELESS. Considering the control subjects, ARNTL (*p* = 0.57), CLOCK (*p* = 0.06), NFIL3 (*p* = 0.06), and PER2 (*p* = 0.128) showed no statistical difference in expression between symptomatic patients and controls, while CRY1 (*p* < 0.001), CRY2 (*p* = 0.011), CSNK1E (*p* = 0.029), DBP (*p* = 0.003), NR1D1 (*p* = 0.027), and SIRT1 (*p* = 0.001) showed a significantly higher expression in symptomatic patients in respect to the controls.

Over-expression (over the 75th percentile of controls) of circadian genes was associated with many significant differences in some clinical and biochemical variables, even if they lingered in their normal ranges. All the significant differences were detailed in Table 5. No gene appeared down-regulated, so we did not test the differences between patients with normal and reduced (under the 25th percentile) expression.

Lower levels of total amylases were found for patients with over-expression of ARTNL, ARTNL2, CLOCK, and CRY2, while lower levels of lipases were found associated with over-expression of CLOCK, CRY1, PER1, PER2, SIRT1, and TEF. ARNTL over-expression was associated also with lower levels of CRP. Some differences in hepatic function markers were found, as higher values of INR were found associated with the over-expression of ARTNL2 and CRY2, higher values of AST with ARTNL2, higher levels of total bilirubin with CLOCK and CSNK1E, higher values of phosphatases with CSNK1E, and higher levels of gamma glutamyl transpeptidase (GGT) were associated with higher expression of PER1. Lower iron blood levels were found in patients with a higher expression of ARNTL2, while higher iron blood levels were found in patients with over-expression of CSNK1E, and higher ferritin values were found in patients with an over-expression of CLOCK.

**Figure 3:**
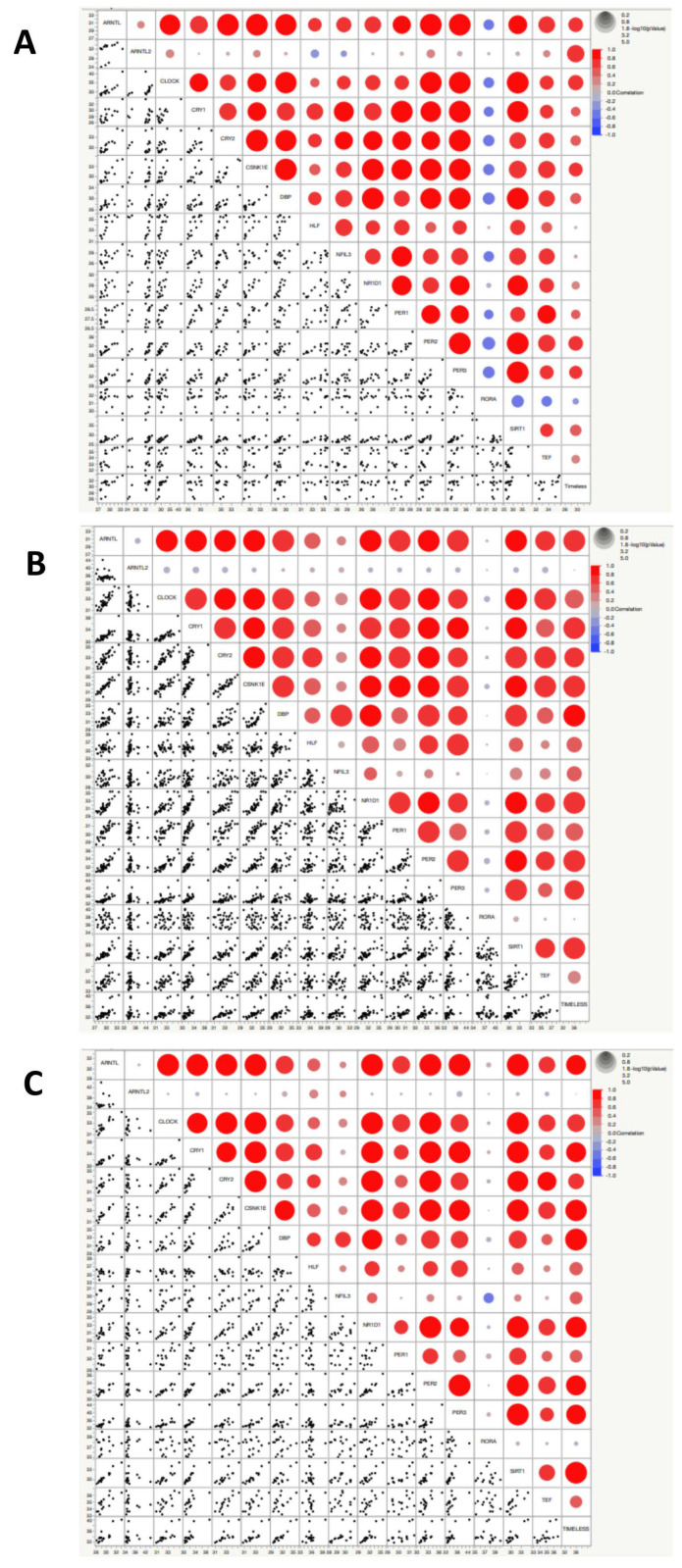
Correlation matrices showing correlation values between the expression of circadian genes in controls (**A**), grouped patients affected by porphyrias (**B**), symptomatic patients affected by porphyrias (**C**). The heat maps show the correlation coefficients, which measure the degree of the linear relationship between each pair of variables. The correlation values can fall between −1 and +1. Positive correlation is measured on a 0.1 to 1.0 scale. The range from 0.1 to 0.3 indicates weak positive correlation, the range from 0.3 to 0.5 indicates a moderate positive correlation, and the range from 0.5 to 1.0 indicates a strong positive correlation. Bubble size represents statistical significance expressed as −Log “*p*-value” (log-rank test), where the larger the size, the greater the significance.

Higher values of alpha-fetoprotein correlated with over-expression of CLOCK and CRY1; lower values of CA 19-9 were found in patients with a higher expression of CLOCK, CRY1 and PER2. Higher values of CA 125 were associated with a higher expression of CRY1 and higher values of CA 15.3 were associated with PER1 over-expression. Lower values of PBG were found in patients with an over-expression of CLOCK and higher values of total porphyrins were found associated with an over-expression of TIMELESS. Higher values of uric acid correlated with over-expression of CRY2, PER2, and TEF. Lower values of chloride were associated with a higher expression of TIMELESS and CSNK1E (associated also with lower values of sodium), while reduced calcium levels were associated with over-expression of PER2. Higher expression of PER1 was associated with no production of anti-thyroid peroxidase (anti-TPO) antibodies. Lower values of urine-specific weight were associated with over-expression of PER2 and TIMELESS, while PER3, SIRT1 and TEF showed an association with higher urine pH values. TIMELESS was also associated with lower levels of urea and parathyroid hormone. Over-expression of DBP, HLF, NFIL3, and NDRD1 was not associated with significant differences in all the tested clinical/biochemical variables. RORA was not tested as all the patients showed an over-expression of this gene. The analysis of the correlation matrices (Figure 3) highlights different profiles when comparing the controls with the global group of patients and even more if only symptomatic patients are considered. In particular, the levels of expression of RORA and TIMELESS show very different correlation patterns compared to the groups of patients, considered globally or only if symptomatic.

## 4. Discussion

Heme is a member of the porphyrins family of cyclic tetrapyrroles, comprising macrocycles composed of four pyrrole-derived rings hinged by methine bridges and undertaking vital roles in various bio-systems. All hemes comprise a central iron ion, which is combined by the four pyrrole nitrogen atoms [37]. In hemoproteins such as hemoglobin and myoglobin, heme is the prosthetic group essential for oxygen transport and storage and is needed in various cytochromes for electron transport and for mixed function oxidases in cytochrome P450. Furthermore, heme is a cofactor of peroxidase for hydrogen peroxide production and of catalase for hydrogen peroxide decomposition. Heme also plays a regulatory role in the sensing of diatomic gases and signal transduction, gene transcription/translation, microRNA processing, protein stability, mitochondrial protein import, metabolic pathways, drug detoxification, and functioning of the molecular clockwork [38]. Defects of heme synthesis can cause various disorders for instance anemia and porphyrias [37]. The levels and activity of enzymes involved in the heme biosynthetic pathway and in particular ALAS1 (5′-Aminolevulinate Synthase 1) show 24-h cyclic fluctuations driven by the molecular clockwork [10]. In turn, heme interacts with REV-ERBs and modulates transcriptional processes in the TTFL, mainly with inhibitory effects on the expression of circadian genes through NCoR-HDAC3 corepressor complex recruitment [29]. Moreover, by means of Rev-erbα–mediated repression of metabolic genes transcription, heme restrains the expression of genes managing gluconeogenesis and glucose output in the liver, so that Rev-erbα works as a heme sensor and harmonizes the biological clock ticking with glucose homeostasis, and energy metabolism [39,40]. Interestingly, in all patients affected by both acute and cutaneous forms of porphyria, heme biosynthesis failure was associated with increased expression levels of the considered circadian genes when compared with the controls, except for ARNTL and TEF. In the patients affected by CEP and PCT the expression levels of DBP and NFIL3 were significantly higher with regard to the patients affected by AIP, while significantly lower expression levels of ARNTL, CLOCK, CRY1, CRY2, CSNK1E, DBP NFIL3, NR1D1, PER2, and SIRT1 were found in symptomatic patients when compared to asymptomatic patients. In the whole group of patients, lower values of PBG were found to be associated with high expression levels of CLOCK and higher values of total porphyrins were found to be associated with high expression levels of TIMELESS. Heme impacts the molecular clockwork binding to different circadian proteins. In particular, heme directly interacts with CLOCK protein in the nucleus of human cells binding to the PAS-A and PAS-B domains, with Histidine residue at position 144 as a ligand, and disrupts CLOCK interactions with the E-boxes in the promoters of target genes, as evidenced by DNA binding assays. Flexibility in the heme pocket scaffolds an additional Histidine and Cystidine coordination and this conformationally mobile CLOCK protein structural framework brings about heme-dependent transcriptional regulation [41]. 

Regarding blood electrolytes balance, lower calcium values were found to be associated with high expression levels of PER2, lower values of chloride and sodium were found to be associated with high expression levels of CSNK1E, and lower values of chloride were found to be associated with high expression levels of TIMELESS. A different pattern of correlation among circadian gene expression levels was found for RORA and TIMELESS expression levels when confronting the controls with the global group of patients and the differences were even more evident when considering exclusively the symptomatic patients. TIMELESS is encompassed in the molecular clockwork and plays a role in embryonic development, cell cycle progression, DNA damage response and cooperates in the replication fork protection complex safeguarding fork integrity and genome stability during DNA replication [42]. 

Our results are partially in agreement with a study performed in six asymptomatic Caucasian postmenopausal women suffering from AIP with and without biochemical activity and four sex-matched controls. The serum levels of cortisol, melatonin, ALA, and PBG were evaluated at 3-h intervals for 21 h and mRNA levels of the circadian genes CRY1, PER2, NR1D1, and genes involved in heme synthesis ALAS1, ALAS2, and PBGD were evaluated in peripheral blood mononuclear cells at 6-h intervals for 24 h. Interestingly, in AIP patients with biochemical activity, the CRY1 expression level was increased and was accompanied by lower serum levels of cortisol with dampened early morning increase and lack of 24-h rhythmicity, suggesting a significant alteration of the circadian clock circuitry [43].

## 5. Conclusions

Overall, the results of our study highlight a global alteration of the expression of circadian genes in the presence of deficits of enzymes involved in the heme biosynthetic pathway. Heme deficiency has direct implications in the pathophysiological characteristics of porphyrias, determining the semeiological and symptomatological picture detectable in the patients suffering from porphyrias. On the other hand, the altered expression of circadian genes involves a widespread modification of metabolic pathways and electrolyte balance that could play a key role in determining the clinical picture found in patients suffering from these rare diseases. In our study, we performed a preliminary investigation to confirm alterations of circadian genes expression in the context of heme synthesis disorders. We analyzed snapshot samples of mRNA extracted from PBMC collected at the same time-of-day, so that the results regarding differential expression of circadian genes are consistent, but we have no time-series data on behavioral patterns or other physiological parameters. Consequently, we cannot make any statement regarding alterations of circadian rhythmicity in patients suffering from porphyrias. We could only hypothesize changes of rhythmic patterns in line with altered clock genes mRNA levels, but this issue could be appropriately addressed in future studies conducted on normal subjects and porphyria patients in a controlled environment and with specific parameters (i.e., temperature, light, nutrition, rest/activity, and segregation). An in-depth knowledge of the mechanisms underlying the altered expression of circadian genes in heme synthesis disorders and a better definition of the pathophysiological alterations consequent to the derangement of the circadian clock circuitry in this context could shed light on important signaling pathways and could represent, in the near future, a therapeutic target in the hope of increasing the effectiveness of pharmacological treatments and improving the quality of life of patients suffering from porphyrias. 

## Figures and Tables

**Table 1:** Laboratory characteristics of patients affected by porphyrias.

Variables	Values	Number
General variables		
BMI (kg/m^2^)	22.9 (19–26)	16
LDH (U/I)	280 (205–300)	10
Creatine kinase (U/I)	62 (41–85)	10
ESR (mm/h)	21 (12–27)	15
CRP (mg/dL)	0.34 (0.32–0.49)	15
Renal function and urinalysis		
GFR CKD/EPI (mL/min)	122 (110–129)	16
Urea (mg/dL)	33.5 (26–45)	16
Creatinine (mg/dL)	0.76 (0.64–0.8)	16
Urine pH	6 (5.6–6.1)	16
Urobilinogen (mg/dL)	0.2 (0–0.7)	16
Nitrates	Negative	15
Urine Specific weight (g/L)	1018 (1014–1021)	16
Urine colture	1 positive for *E. coli*	15
Porphyrins and other metabolites		
ALA (mg/L)	2.4 (1.8–7.1)	16
PBG (umol/L)	1.4 (1–13.6)	16
Total porphyrins (ug/24 h)	158 (104–493)	15
Fecal porphyrins (mcg/24 h)	140 (45–33,000)	7
Uroporphyrins (mcg/24/h)	4000 (5–8100)	4
Fecal coproporphyrins (ug/L)	3200 (47–7000)	4
Thyroid Function		
FT3 (nmol/L)	3.2 (2.9–3.6)	15
FT4 (nmol/L)	1.3 (1–1.4)	15
TSH (umol/mL)	1.5 (1.1–2.2)	15
Anti-TG (IU/mL)	9.6 (0.1–16)	15
anti-TPO (IU/mL)	3.9 (0.2–22.5)	15
Coagulation		
INR	1 (1–1.1)	16
Fibrinogen (mg/dL)	300 (260–340)	7
D-dimer (ng/mL)	270 (74–380)	13
Metabolism		
Blood glucose (mg/dL)	86 (64–91)	16
Uric acid (mg/dL)	4.3 (3.6–91)	16
Total serum proteins (g/dL)	7.2 (6.8–7.5)	16
Albumin (g/dL)	4.3 (4.1–4.5)	5
Total cholesterol (mg/dL)	188 (164–209)	16
LDL cholesterol (mg/dL)	99 (85–140)	9
HDL cholesterol (mg/dL)	58 (41–63)	10
Triglycerides (mg/dL)	90 (68–135)	16
Glycated Hemoglobin (%)	5.5 (5.4–5.9)	4
Vitamin D (ng/mL)	7 (3.6–15)	5
Electrolytes		
Sodium (mEq/L)	141 (139–141)	16
Chloride (mEq/L)	105 (104–107)	16
Potassium (mEq/L)	4.3 (4.1–4.4)	16
Phosphorus (mg/dL)	3.3 (3.1–3.7)	16
Magnesium (mEq/L)	2.2 (2.1–2.2)	5
Calcium (mEq/L)	8.9 (8.6–9.4)	16
Hepatic and pancreatic function		
Total bilirubin (mg/dL)	0.5 (0.5–0.7)	16
Alanine aminotransferase (U/I)	26 (20–32)	16
Aspartate aminotransferase (U/I)	25 (20–44)	16
GGT (U/L)	21 (14–32)	16
Alkaline phosfatase (U/L)	71 (62–93)	16
Amylases (U/I)	63 (54–76	12
Lipases (U/I)	33 (25–50)	8
HbsAg	1 positive	15
anti-HCV antibodies	1 positive	15
Blood cell counts		
Hemoglobin (g/dL)	13.6 (12.3–14.5)	16
White blood cells (U/L)	6600 (5700–7700)	16
Platelets (U/I)	236,000 (172,000–256,000)	16
Iron metabolism		
Serum iron (mcg/dL)	78 (73–94)	16
Ferritin (ng/mL)	84 (31–143)	16
Transferrin (mg/dL)	231 (211–271)	16
Tumor markers		
Alphafetoprotein (ng/mL)	2.7 (2.3–3.4)	16
CEA (ng/mL)	2 (1–4)	16
Ca 19.9 (U/mL)	10.5 (2.2–16.8)	16
Ca 15.3 (U/mL)	13.1 (0.7–18)	10
Ca 125 (U/mL)	17.6 (9.4–18.4)	13
PTH (pmol/mL)	48 (26–88)	14

The numbers in parentheses in the values column are the detected value ranges. The third column reports the number of subjects evaluated for each variable. BMI = Body mass index; LDH = lactate dehydrogenase; ESR = erythrocyte sedimentation rate; CRP = C-Reactive Protein; GFR = glomerular filtration rate; CKD-EPI = Chronic Kidney Disease Epidemiology Collaboration); ALA = Aminolevulinic acid; PBG = porfobilinogen; FT3 = i free triiodothyronine; FT4 = free thyroxine; TSH = Thyroid-stimulating hormone; Anti-TG = Anti-thyroglobulin antibodies; anti-TPO = Anti-thyroid peroxidase antibodies; INR = International Normalized Ratio; LDL = low-density lipoprotein; HDL = high-density lipoprotein; GGT = gamma-glutamyl transferase; CEA = carcinoembryonic antigen; PTH = parathyroid hormone.

**Table 2:** Genes and proteins mutations found in the enrolled patients affected by porphyrias.

Mutated Gene	Gene Mutation	Protein Mutation	Mutation Type	
*HMBS*	*HMBS* c.181delG		frameshift	
*HMBS*	*HMBS* c.72_77delTACCCG	p.Thr25_Arg26del	p.del	
*HMBS*	*HMBS* c.772-3C>G		splicing	
*HMBS*	*HMBS* c.652-2delA		frameshift	
*HMBS*	*HMBS* c.580C>T	p.Q194X	nonsense	
*CPOX*	*CPOX* c.148C>T	p.50	missense	new
*CPOX*	*CPOX* c.395C>T	p.132A>V	missense	new
*CPOX*	*CPOX* c.613G>T	p.205V>L	missense	new
*UROD*	*UROD* C.1000A>G	p.394I>V ?	missense	
*UROD*	*UROD* c.246-248delCAT	p.del		new
*UROS*	*UROS* c.660+4delA		splicing	new

**Table 3:** Circadian genes expression in controls and patients affected by porphyrias and differences between the groups.

Genes	Values (Medians and IQRs)	*p* Value
ARNTL		0.647
	
*Controls*	28.8 (28–30)	
AIP	29.5 (28.7–30.3)	
HCP	29.3 (28.7–30.3)	
CEP + PCT	30 (29–31.4)	
ARNTL2		**<0.001**
	
*Controls*	33 (32–33.7)	
AIP	35 (34.7–35.5)	
HCP	35.4 (35–38)	
CEP + PCT	35.5 (34.8–39.8)	
CLOCK		**0.006**
	
*Controls*	30.3 (29.1–32)	
AIP	32.2 (31.8–33.3)	
HCP	32.7 (32.2–33.7)	
CEP + PCT	33 (31.6–33.7)	
CRY1		**<0.001**
	
*Controls*	28 (26.7–29.7)	
AIP	31.8 (30.9–32.3)	
HCP	32 (31.5–32.3)	
CEP + PCT	32.5 (30.6–33.7)	
CRY2		**0.002**
	
*Controls*	29.8 (29–31.5)	
AIP	32.7 (32–33.7)	
HCP	32.5 (32.2–33.2)	
CEP + PCT	32.9 (32.2–33.9)	
CSNK1E		**0.009**
	
*Controls*	29.8 (28.7–31.1)	
AIP	32.5 (31.7–33.1)	
HCP	32.6 (31.9–33.3)	
CEP + PCT	33.1 (31.9–34.3)	
DBP		**<0.001**
	
*Controls*	27.8 (27–29.1)	
AIP	31.1 (30.1–31.8)	
HCP	30.9 (30.4–32)	
CEP + PCT	32.9 (31.8–34)	
HLF		**0.013**
	
*Controls*	34.6 (32.3–35.4)	
AIP	35.6 (35–36.2)	
HCP	35.8 (35.4–36.2)	
CEP + PCT	35.4 (35.4–35.9)	
NFIL3		**0.001**
	
*Controls*	28.1 (26.3–29.1)	
AIP	28.9 (28.5–29.7)	
HCP	29.7 (29.2–30.2)	
CEP + PCT	30.6 (29.7–31.2)	
NR1D1		**<0.001**
	
*Controls*	27.4 (26.2–28.5)	
AIP	31.2 (30.5–32.2)	
HCP	31.1 (30.1–31.9)	
CEP + PCT	32.3 (30.8–33.5)	
PER1		**<0.001**
	
*Controls*	27.9 (27.2–28.6)	
AIP	30.3 (29.6–30.7)	
HCP	30.8 (30.1–30.9)	
CEP + PCT	30.3 (29.5–31.1)	
PER2		**0.032**
	
*Controls*	29.9 (29.1–32.4)	
AIP	32.1 (31.6–33.6)	
HCP	32.4 (32.2–32.7)	
CEP + PCT	32.2 (31.6–33.9)	
PER3		**0.001**
	
*Controls*	30.9 (30–32.5)	
AIP	33.8 (33.2–35.2)	
HCP	34 (33.6–34.8)	
CEP + PCT	34.4 (33–37)	
RORA		**<0.001**
	
*Controls*	31.8 (31.5–32.2)	
AIP	37.4 (35.5–38.2)	
HCP	36.7 (35.6–38.5)	
CEP + PCT	37 (36.1–37.8)	
SIRT1		**<0.001**
	
*Controls*	27.5 (26.3–28.5)	
AIP	30.2 (29.7–30.5)	
HCP	30.5 (29.6–31.2)	
CEP + PCT	30.8 (29.3–33.9)	
TEF		0.21
	
*Controls*	33.3 (32.7–35.3)	
AIP	34.8 (34–35.3)	
HCP	34.8 (34.2–35.2)	
CEP + PCT	35 (33.8–36.4)	
TIMELESS		**<0.001**
	
*Controls*	31.3 (29.5–31.7)	
AIP	33.1 (32.2–33.5)	
HCP	33.3 (33.1–34.3)	
CEP + PCT	34 (33.5–39.6)	

The numbers in parentheses in the values column are the detected value ranges.

**Table 4:** Differences in circadian genes expression between patients with signs and symptoms of porphyrias and asymptomatic porphyria patients.

Gene	Asymptomatic Patients	Symptomatic Patients	*p* Value
ARNTL	30 (29–30.7)	28.7 (28.6–29)	**0.006**
ARNTL2	35.4 (34.7–35.5)	34.8 (34.2–35.9)	0.38
CLOCK	33.3 (31.9–33.7)	31.9 (31.4–32.3)	**0.027**
CRY1	32.1 (31.7–33.1)	31 (30.8–31.8)	**0.017**
CRY2	33.2 (32.3–34.1)	32.2 (31.6–32.3)	**0.02**
CSNK1E	33 (32–34)	31.9 (31–32.3)	**0.023**
DBP	31.8 (30.7–33.3)	30.4 (30–31)	**0.027**
HLF	35.6 (35.3–35.9)	35.7 (35.1–36)	0.88
NFIL3	30 (29.5–30.9)	29 (28–30)	**0.049**
NR1D1	31.9 (31–33)	30.9 (30–31)	**0.027**
PER1	30.8 (29.5–31.2)	30.1 (29.6–30.3)	0.14
PER2	32.5 (31.7–33.8)	30.1 (29.6–30.3)	**<0.001**
PER3	35 (33.4–35.9)	33.4 (33.1–33.9)	0.079
RORA	37 (36–38.3)	38 (35.5–38.5)	0.77
SIRT1	31.1 (30–32)	29.7 (29–30)	**0.008**
TEF	35 (34.4–35.7)	34 (33.6–34.8)	0.062
TIMELESS	33.5 (33.2–34.4)	33.1 (32.3–33.6)	0.16

The numbers in parentheses in the values column are the detected value ranges.

**Table 5:** Laboratory characteristics found significantly different between porphyria patients with normal expression and porphyria patients with over-expression of circadian genes.

Genes and Variables	Normal Expression	Over-Expression (>75th Percentile)	*p* Value
ARTNL			
Amylases (U/I)	67 (61–80)	52 (37–55)	0.033
CRP (mg/dL)	0.36 (0.33–0.9)	0.32 (0.30–0.33)	0.049
ARTNL2			
Amylases (U/L)	76 (67–89)	58 (46–61)	0.025
AST (U/L)	30 (20–33)	86 (84–95)	0.033
INR	1 (0.9–1)	1.1 (1–1.1)	0.049
Iron blood level (mcg/dL)	87 (77–104)	73 (64–80)	0.034
CLOCK		3.3 (2.7–4)	0.007
Alpha-fetoprotein (ng/mL)	2.2 (1.45–2.4)	54 (46–61)	0.02
Amylases (U/L)	73 (65–89)	0.6 (0.49–0.83)	0.039
Total bilirubin (mg/dL)	0.39 (0.38–0.51)	3 (1–14)	0.026
Ca 19.9 (U/mL)	16 (10–20)	25 (20–33)	0.049
Lipases (U/L)	50 (37–58)	118 (86–182)	0.49
Ferritin (ng/mL)	32 (22–66)	1.1 (0.9–1.6)	0.044
PBG (umol/L)	9.7 (1–47)		
CRY1			
Alpha-fetoprotein (ng/mL)	2 (1.4–2.4)	3.3 (2.6–3.8)	0.015
Ca 125 (U/mL)	18 (15–18.5)	22 (19–25)	0.037
Ca 19.9 (U/mL)	16 (12–20)	3.4 (1–13)	0.045
Lipases (U/L)	52 (47–63)	26 (22–30)	0.025
CRY2			
Uric acid (mg/dL)	3.8 (3.6–4.2)	4.5 (4.3–5.2)	0.039
Amylases (U/L)	73 (65–105)	55 (48–66)	0.034
INR	1 (0.9–1.1)	1.1 (1–1.1)	0.032
CSNK1E			
Total bilirubin (mg/dL)	0.039 (0.38–0.57)	0.6 (0.5–0.8)	0.044
Chloride (mEq/L)	107 (104–108)	104 (100–105)	0.049
Alkaline phosphatase (U/L)	70 (56–72)	93 (73–110)	0.039
Iron blood level (mcg/dL)	75 (64–78)	96 (81–104)	0.011
Sodium (mEq/L)	141 (140–142)	140 (135–140)	0.015
*PER1*			
*Ca 15.3 (U/mL)*	9.6 (8.8–10.4)	18 (15–25)	0.014
*GGT (U/I)*	13.5 (11.5–17)	23.5 (21–37)	0.039
*Lipases (U/I)*	52 (47–63)	26 (22–30)	0.025
*anti-TPO (IU/mL)*	23 (9–28)	0.15 (0.1–7)	0.042
PER2			
Uric acid (mg/dL)	3.9 (3.2–4.5)	4.5 (4–4.5)	0.047
Ca 19.9 (U/mL)	16 (8–19)	3 (1–11)	0.044
Calcium (mEq/L)	9.4 (8.8–9.5)	8.6 (8.4–8.9)	0.02
Lipases (U/L)	44 (26–52)	20 (17–23)	0.046
Urine specific weight (g/L)	1019 (1016–1024)	1014 (1011–1017)	0.039
PER 3			
Urine pH	5.6 (5.5–5.8)	6 (5.9–6.5)	0.016
SIRT1			
Lipases (U/L)	50 (37–58)	25 (20–33)	0.048
Urine pH	5.6 (5.5–5.8)	6.1 (6–6.5)	0.012
TEF			
Uric Acid (mg/dL)	3.8 (3.3–4.3)	4.5 (4.4–4.9)	0.047
Lipases (U/I)	47 (37–55)	23 (17–26)	0.037
Urine pH	5.6 (5.5–5.8)	6.2 (6–6.7)	0.004
*TIMELESS*			
*Chloride (mEq/L)*	107 (106–109)	105 (102–106)	0.027
*Urine specific weight (g/L)*	1022 (1017–1027)	1015 (1013–1019)	0.031
*Total porphyrins (ug/mL)*	87 (67–162)	233 (134–937)	0.037
*Urea (mg/dL)*	46 (40–51)	30 (21–34)	0.041
*PTH (pmol/mL)*	88 (59–95)	37 (24–49)	0.046

The numbers in parentheses in the values column are the detected value ranges. CRP = C-Reactive Protein; PBG = porfobilinogen; AST = Aspartate aminotransferase; anti-TPO = Anti-thyroid peroxidase antibodies; INR = International Normalized Ratio; GGT = gamma-glutamyl transferase; PTH = parathyroid hormone.

## Data Availability

Data will be made available on request.

## Supplementary Materials

The following supporting information can be downloaded at: https://www.mdpi.com/article/10.3390/biomedicines10123198/s1. Table S1: List of human QuantiTec Primers used for RT-PCR.
