# Supplementary material for: Circadian Genes Expression Patterns in Disorders Due to Enzyme Deficiencies in the Heme Biosynthetic Pathway
Source: Biomedicines. 2022 Dec 9;10(12):3198. doi: 10.3390/biomedicines10123198 (PMC9775071; doi:10.3390/biomedicines10123198)
Supplement: Supplementary file 1 [file biomedicines-10-03198-s001.zip › biomedicines-2059056-supplementary.pdf]

Supplementary Table S1. List of human QuantiTec Primers used for RT-PCR
Hs_ARNTL_1_SG QuantiTect Primer Assay (QT00011844)
Hs_ARNTL2_1_SG QuantiTect Primer Assay (QT00068250)
Hs_CLOCK_1_SG QuantiTect Primer Assay (QT00054481)
Hs_CRY1_1_SG QuantiTect Primer Assay (QT00025067)
Hs_CRY2_1_SG QuantiTect Primer Assay (QT00094920)
Hs_CSNK1E_1_SG QuantiTect Primer Assay (QT00999152)
Hs_DBP_1_SG QuantiTect Primer Assay (QT00055755)
Hs_HLF_1_SG QuantiTect Primer Assay (QT00031752)
Hs_NFIL3_1_SG QuantiTect Primer Assay (QT00013944)
Hs_NR1D1_1_SG QuantiTect Primer Assay (QT00000413)
Hs_PER1_1_SG QuantiTect Primer Assay (QT00069265)
Hs_PER2_1_SG QuantiTect Primer Assay (QT00011207)
Hs_PER3_1_SG QuantiTect Primer Assay (QT00097713)
Hs_RORA_1_SG QuantiTect Primer Assay (QT00072380)
Hs_SIRT1_1_SG QuantiTect Primer Assay (QT00051261)
Hs_TEF_1_SG QuantiTect Primer Assay (QT00032732)
Hs_TIMELESS_1_SG QuantiTect Primer Assay (QT00019789)
